# Effects of Ultrafine Particles in Ambient Air on Primary Health Care Consultations for Diabetes in Children and Elderly Population in Ljubljana, Slovenia: A 5-Year Time-Trend Study

**DOI:** 10.3390/ijerph17144970

**Published:** 2020-07-10

**Authors:** Vesna Viher Hrženjak, Andreja Kukec, Ivan Eržen, Dalibor Stanimirović

**Affiliations:** 1 National Laboratory of Health, Environment and Food, Prvomajska 1, 2000 Maribor, Slovenia; vesna.viher.hrzenjak@nlzoh.si; 2 Faculty of Medicine, University of Ljubljana, Vrazov trg 2, 1000 Ljubljana, Slovenia; andreja.kukec@mf.uni-lj.si (A.K.); ivan.erzen@nijz.si (I.E.); 3 National Institute of Public Health, Trubarjeva 2, 1000 Ljubljana, Slovenia

**Keywords:** air pollution, ultrafine particles, diabetes, health effects, time-trend study, Slovenia

## Abstract

Epidemiological studies indicate that exposure to ultrafine particles (UFP) in ambient air represents an important environmental public health issue. The aim of this study was to determine the association between UFP in ambient air and the daily number of consultations in the primary health care unit due to diabetes mellitus in children and elderly population of the Municipality of Ljubljana. A 5-year time-trend ecological study was carried out for the period between 1 January 2013 and 31 December 2017. The daily number of primary health care consultations due to diabetes mellitus among children and elderly population was observed as the health outcome. Daily mean UFP concentrations (different size from 10 to 100 nm) were measured and calculated. Poisson regression analysis was used to investigate the association between the observed outcome and the daily UFP, particulate matter fine fraction (PM_2.5_), and particulate matter coarse fraction (PM_10_) concentrations, adjusted to other covariates. The results show that the daily number of consultations due to diabetes mellitus were highly significantly associated with the daily concentrations of UFP (10 to 20 nm; *p* ≤ 0.001 and 20 to 30 nm; *p* ≤ 0.001) in all age groups and in the elderly population. In observed the population of children, we did not confirm the association. Findings indicate that specified environmental challenges should be addressed by comprehensive public health strategies leading to the coordinated cross-sectoral measures for the reduction of UFP in ambient air and the mitigation of adverse health effects.

## 1. Introduction

Air pollution and diabetes mellitus are critical public health issues worldwide [1]. Air pollution is a problem that is faced by all societies at all levels of development; especially at risk is the urban population [2]. Air pollution leads to 8.8 million premature deaths each year globally [3] and is the second leading course of deaths from noncommunicable diseases, including especially ischaemic heart disease, stroke, chronic obstructive pulmonary disease, and lung cancer [4]. Evidence is emerging on possible links between air pollution and other health outcomes, such as neurodevelopmental disorders, cognitive impairment and chronic disease conditions, such as diabetes [5]. Diabetes is a metabolic disorder caused by genetic and environmental factors, which lead to insufficient insulin secretion. The prevalence of diabetes has rapidly increased over the last few decades, and it is considered as one of the major contributors to the global burden of disease and premature deaths [6,7,8,9].

Currently, the most recognized adverse effects of air pollution on human health are due to the action of particulate matter (PM), nitrogen dioxide (NO_2_), and ground-level ozone (O_3_) [10]. PM, mainly its fine fraction (PM_2.5_), is a prime public health concern [4]. PM health effects have been investigated in many studies. Results from original studies have also been combined in reviews and meta-analyzes [11,12,13,14,15,16,17]. These studies have found that PM in ambient air has been linked to all-cause mortality, cardiovascular diseases, and respiratory diseases. Some population groups are more affected by air pollution than others, such as older people, children, and those with pre-existing health conditions, who are more vulnerable. Toxicological evidence suggests that PM may lead to adverse effects on human health through inflammation in the respiratory or circulatory system, systemic oxidative stress, or by activating the autonomic nervous system [18]. Over the last few years, a growing number of toxicological studies have also suggested an association between PM_2.5_ and diabetes through an inflammatory pathway leading to endothelial dysfunction, immune response alterations in visceral adipose tissues, and endoplasmic reticulum stress leading to alterations in insulin transduction, insulin sensitivity, and glucose metabolism [19,20,21,22,23]. A growing number of epidemiological studies have been trying to explore the associations between particulate air pollution and diabetes, and also some reviews and meta-analyzes concerning this topic have been published [1,24,25,26,27,28]. Several of them concluded that air pollution is likely a risk factor for diabetes. Current cumulative evidence appears to suggest that type 2 diabetes mellitus-related biomarkers increase with increasing exposure duration and concentrations of air pollutants [1]. However, the results from diverse epidemiological studies are not fully consistent, and no systematic reviews have yet been published to determine whether these claims are substantiated and verified.

In the toxicity of particles, size and morphology play a key role. Division of particle size most commonly draws a distinction between coarse and fine particles—fine particles (PM_2.5_) are smaller than 2.5 µm (AED ≤ 2.5 μm), and coarse particles (PM_10_) are larger (AED ≤ 10 μm). Ultrafine particles (UFP) are defined as particles smaller than 0.1 μm (AED ≤ 0.1 μm) [29]. In the particle measurements, they are the prime contributor to the number concentrations with more than 80% of the total airborne particle number in urban and industrial environments, while they contribute little to the mass [29,30,31]. They are mainly generated by road traffic, the burning of fossil fuels and biomass, and by industrial emissions, but can also originate from natural sources [29,32,33,34].

Due to their small size, UFP are believed to exert higher toxicity than larger particles. UFP have a higher surface area per unit mass. Large relative surface area absorbs relatively higher amounts of toxic materials increasing the exchange of toxic material with the physiological medium. The lower aerodynamic diameter of UFP allows them to penetrate into the deepest parts of the respiratory tract (alveoli) where there are no flagella, and where the exchange of gases between the inhaled air and the blood takes place. They are eliminated less efficiently from the respiratory tract, which increases their residence time and facilitates their accumulation. After that, they cross the alveolar barrier, enter the bloodstream, and they can travel throughout the body, passing even through the placenta [29,35,36].

A growing number of studies evaluate the role of UFP exposure on adverse health outcomes. Most of what we know about possible health effects from UFP exposure comes from research done on animals [29]. Strong and consistent evidence from animal studies indicates long-term exposure to UFP is related to negative effects on the brain, nervous, and respiratory system [29,37,38,39,40,41,42,43,44,45,46,47,48,49,50,51,52,53,54,55]. A few animal studies have reported effects on reproduction and development [29,56,57,58,59,60]. The body of epidemiological literature on the association between health and UFP exposure is limited but growing [29,61,62,63]. Increasing evidence suggests UFP can cause adverse health outcomes, especially on respiratory and cardiovascular morbidity and mortality. The evidence suggests adverse short-term associations with inflammatory and cardiovascular changes, which may be at least partly independent of other pollutants [62], but it is not clear whether short- and long-term exposure in humans can induce adverse health effects to the same extent as those observed in animals [32,36]. For other studied health outcomes, such as the diabetes mellitus, the evidence on independent health effects of UFP remains inconclusive or insufficient [62], which is related to the fact that actual data on UFP exposure are rather undependable. The aim of our study was to determine the association between UFP in ambient air and the daily number of consultations in the primary health care unit due to diabetes mellitus in children and elderly population of the municipality of Ljubljana (MOL), adjusted for other co-pollutants, seasonal, and meteorological factors. 

## 2. Materials and Methods

### 2.1. Study Design, Period, and Area of Observation

Ecological time-trend design was used to assess the association between UFP in ambient air and the daily number of consultations in the primary health care unit due to diabetes mellitus in children and the elderly population of MOL. The unit of observation was a single day of the observed period between 1 January 2013 and 31 December 2017.

**Figure 1:**
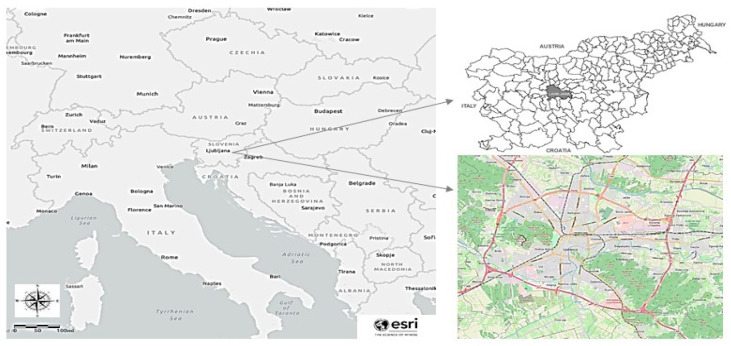
A map of Slovenia surrounded by bordering countries as well as the location of the capital city Ljubljana with the borders of the municipality, hydrography, and roads.

The study was carried out in an administrative unit of the MOL, which is the largest city and capital of Slovenia, with a population between approximately 282,741 and 288,250 people over the period 2013–2017 (Figure 1). It has an area of 275 km^2^ and its population density varies from 1028.1 to 1048.2 people per km^2^ over the period 2013–2017 [64]. It is located at 46°03′20″ N 14°30′30″ E and has an average elevation of 295 m. It has a continental climate (bordering on a subtropical humid climate), which is characterized by moderately cold winters and warm summers. The average yearly temperature in 2018 was 12.5 °C (January 2018 average: 4.8 °C and July 2018 average: 22.3 °C). Yearly precipitation in 2018 was approximately 1400 mm and the average annual percentage of humidity was 75.0%. The economic activity in the MOL is very dynamic, with around 50,000 companies operating mainly in the pharmaceutical, petrochemical, and food industries. Other economic activities include banking, finance, transport, construction, crafts, services, and tourism. Due to air quality perspective, MOL is a highly polluted municipality in Slovenia with PM_10_. The reasons are geographical characteristics (poorly ventilated basin), meteorological conditions (temperature inversions are frequently present in winter and autumn season), and largely road transport factors, especially in its narrower urban area with heavy traffic [65].

### 2.2. Observed Population 

The observed population consisted of all individuals, residing in MOL temporarily and permanently, who visited the primary health care units of the Community Health Center (CHC) Ljubljana due to diabetes mellitus in the period of observation.

### 2.3. Data Collection

#### 2.3.1. Observed Health Outcomes

Routinely collected health data were obtained from the health information system of the CHC Ljubljana. The daily number of primary health care consultations due to diabetes mellitus (WHO International Classification of Diseases, version 10: E10-E14) [66] among all observed populations, children (from birth to 10 years old) and the elderly (60 year +) population, were observed as the health outcomes.

#### 2.3.2. Observed UFP and PM Data and Meteorological Data

Within the project “Ultrafine particles—an evidence based contribution to the development of regional and European environmental and health policy” (UFIREG) UFP number concentrations (PNC) measurements in different size ranges from 10 until 800 nm using custom-made mobility particle size spectrometers in five cities, including Ljubljana, were conducted. In Ljubljana UFP number concentrations were collected on a measuring site located in the urban environment of MOL (Hajdrihova street 19, Ljubljana). Kindergarten, primary school and multistory buildings were situated in the vicinity of the measuring site. The measuring site was situated 50 m from the nearest low-frequented road, but it was not in the direct vicinity of highly-frequented roads. The railway station is situated near the measuring site (approximately 1 km). The measuring site was considered as representative for the substantial part of the population of MOL by the UFIREG study group. The project started in July 2011 and ended in December 2014 [67]. After the end of the project UFP number concentrations measurements in Ljubljana were continued for another 5 years. UFP number concentrations were measured in 5 min time intervals and then calculated to hourly and daily average concentrations for five size ranges: UFP size from 0.01 to 0.02 μm, from 0.02 to 0.03 μm, from 0.03 to 0.05 μm, from 0.05 to 0.07 μm, and from 0.07 to 0.10 μm as particle number/cm^3^. For this study, data on daily average concentrations for the outlined five size ranges between 1 January 2013 and 31 December 2017 were used.

Daily average PM_10_ and PM_2.5_ concentrations (in µg/m^3^), the daily mean value of air temperature (in °C), and relative humidity (as %) were obtained at the fixed measuring station in Ljubljana, which is a part of the National automated network for monitoring air quality. The data were obtained in agreement with the Slovenian Environment Agency [68]. 

#### 2.3.3. Data on Seasonal Factors and Pollen Concentration

The following seasonal factors were considered in the analysis: season of the year (spring, summer, autumn, winter), working day (yes-weekday/no-weekend), and influenza season (yes/no). Data on influenza season were obtained from annual reports (Epidemiological surveillance of communicable diseases in Slovenia) of the National Institute of Public Health of the Republic of Slovenia [69].

Data on pollen concentration (grains of pollen/m^3^ over a 24-h period) of different allergenic plants (cypress family, grass family, ragweed, birch) were obtained from measuring the station in Ljubljana for the period of observation. The data were acquired from a 2-h collection of pollen concentrations from the database located at the National Laboratory of Health, Environment and Food [70].

### 2.4. Statistical Analysis

#### 2.4.1. Data Description

The distributions of observed health outcomes, selected air pollution data, and meteorological parameters were statistically described by non-parametric typical statistical values (mean, standard deviation, minimum, maximum, 1st, median, and 3rd quartile). The temporal variability of observed health outcomes, selected air pollution data and pollen concentration were presented in sequence charts [71].

#### 2.4.2. Correlation Analysis

The Pearson correlation coefficient was used to assess the ecological correlation a) between exposure (daily average (24-h) UFP (five size ranges), average 24-h PM_10_ and PM_2.5_ concentrations), and outcome variables (daily number of consultations due to diabetes mellitus, separate for three age groups—all age groups, children, and the elderly), and b) between the daily average 24-h number concentration of UFP and the average 24-h concentration of PM_10_ and PM_2.5_ and observed meteorological conditions.

#### 2.4.3. Temporal Relationship Analysis

In the temporal relationship analysis, the daily number of consultations due to diabetes mellitus (separate for all age groups, children, and the elderly) were considered as the observed health outcomes. As the explanatory factors, the daily average 24-h number concentration of UFP and the average 24-h concentration of PM_10_ and PM_2.5_ were considered. Data on seasonal factors, pollen concentration, and meteorological parameters were considered as covariates. Respectively, lags from zero up to five days from exposure to the consultation were examined to determine the amount of time between exposure and effect for observed pollutants in ambient air and pollen data. 

Poisson regression analysis was used to investigate the association between the observed outcome and the daily UFP, PM_2.5_, and PM_10_ concentrations, adjusted to other covariates. Poisson regression is a regression technique used when the outcome is a count variable or a rate [72].

The modelling procedure was performed in two stages. In the first stage, non-adjusted models were built by only relating the observed outcomes and explanatory factors. In the second stage, adjusted models were built by adding the explanatory factor to a core covariate model that included seasonal parameters, meteorological data, and pollen data. The interpretable end-result was the incidence rate ratio (IRR). It was presented together with its lower and upper 95% confidence interval (CI). A *p*-value of 0.05 or less was considered as statistically significant in all statistical tests. All statistical analyses were carried out by using the SPSS software platform, version 25.0 (SPSS Inc., Chicago, IL, USA).

The protocol of the study was approved by the National Medical Ethics Committee (0120-109/2017-5 KME 71/03/17, 2017). In addition, consent from the Community Health Center Ljubljana for using data was obtained (820-1-4/2017, 2017). All data were depersonalized and provided for use in this study in an aggregated form. 

## 3. Results

### 3.1. Data Description

**Figure 2:**
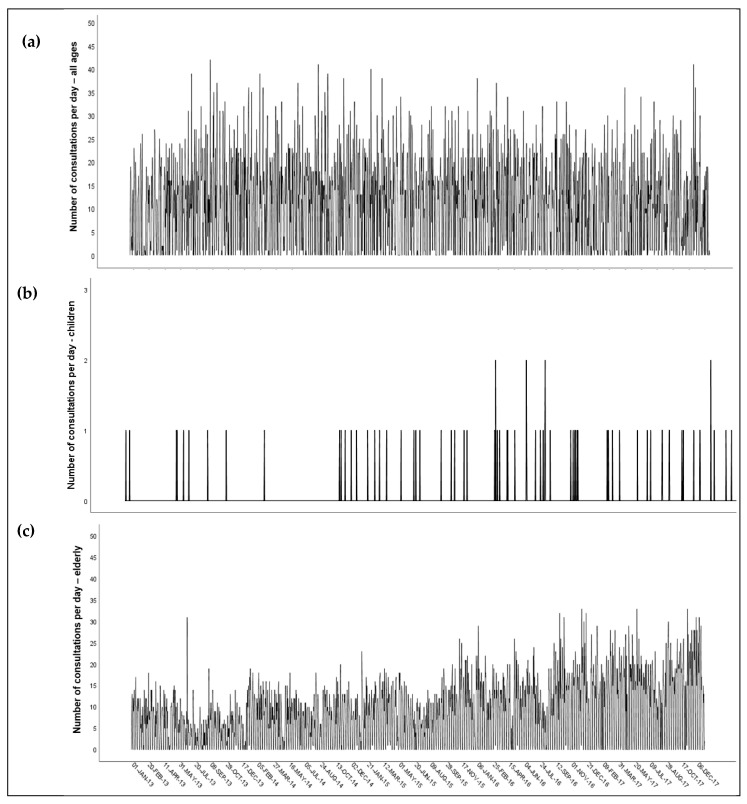
Temporal variability of the daily number of consultations due to diabetes mellitus: (**a**) all age groups; (**b**) children; (**c**) the elderly in the MOL, Slovenia between 1 January 2013 and 31 December 2017.

The daily number of consultations due to diabetes mellitus: all age groups, children, and the elderly were available for all 1.826 days of the study period. There were 373/1.826 (20.4%) days with no consultations for observed health outcomes. More detailed information is available in Table 1. The temporal variability of daily consultations for observed health outcomes is presented with sequence diagrams in Figure 2a–c.

**Figure 3:**
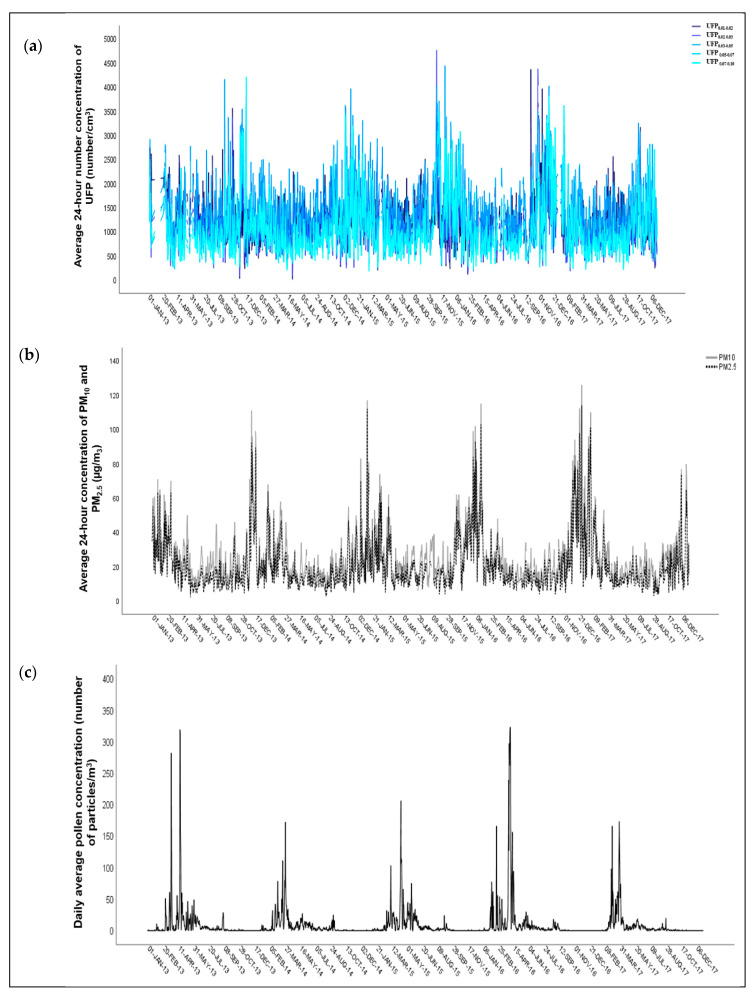
Temporal variability of: (**a**) average 24-h number concentration of UFP (number/cm^3^); (**b**) average 24-h concentration of PM_10_ and PM_2.5_ (µg/m^3^); (**c**) daily average pollen concentration (number of particles/m^3^) in the MOL, Slovenia between 1 January 2013 and 31 December 2017.

The average 24-h number concentration of UFP data for individual size ranges (UFP_0.01–0.02_, UFP_0.02–0.03_, UFP_0.03–0.05_, UFP_0.05–0.07_, and UFP_0.07–0.10_) in the observed period was measured for 1.655/1.826 (90.6%) days. The average 24-h concentration of PM_10_ and PM_2.5_ in the observed period was available for 1.765/1.826 (96.6%) days and for 1.742/1.826 (95.4%) days. More detailed information is available in Table 1. Temporal variability of daily UFP and PM_10 and 2.5_ data is presented with sequence diagrams in Figure 3a,b. In all size ranges of UFP and PM_10 and 2.5_, the daily number of concentrations were highest in the winter season.

Complete data for the daily meteorological factors (air temperature and relative humidity) were available for all 1.826 days of the study period (Table 1). Daily average pollen concentration was the highest in the spring season (Figure 3c).

### 3.2. Correlation Analysis

The Pearson correlation coefficient between average 24-h UFP_0.01–0.02_ concentrations and daily number of consultations due to diabetes mellitus in the total observed population (separate for all age groups, children, and the elderly) ranged from 0.033 to 0.212. With the exception of children (*p* = 0.183), a statistically significant correlation was confirmed for all age groups and the elderly (*p* ≤ 0.001).

The Pearson correlation coefficient between average 24-h UFP_0.02–0.03_ concentrations and the daily number of consultations due to diabetes mellitus in the total observed population (separate for all age groups, children, and the elderly) ranged from 0.047 to 0.254. With the exception of children (*p* = 0.058), a statistically significant correlation was confirmed for all age groups and the elderly (*p* ≤ 0.001).

The Pearson correlation coefficient between average 24-h UFP_0.03–0.05_ concentrations and daily number of consultations due to diabetes mellitus in the total observed population (separate for all age groups, children, and the elderly) ranged from 0.054 to 0.265. A statistically significant correlation was confirmed for all age groups, the elderly (*p* ≤ 0.001), and children (*p* = 0.029).

The Pearson correlation coefficient between average 24-h UFP_0.05–0.07_ concentrations and the daily number of consultations due to diabetes mellitus in the total observed population (separate for all age groups, children, and the elderly) ranged from 0.034 to 0.187. With the exception of children (*p* = 0.171), a statistically significant correlation was confirmed for all age groups and the elderly (*p* ≤ 0.001).

The Pearson correlation coefficient between average 24-h UFP_0.07–0.10_ concentrations and the daily number of consultations due to diabetes mellitus in the total observed population (separate for all age groups, children, and the elderly) ranged from 0.010 to 0.114. With the exception of children (*p* = 0.688), a statistically significant correlation was confirmed for all age groups and the elderly (*p* ≤ 0.001).

The Pearson correlation coefficient between average 24-h PM_2.5_ concentrations and the daily number of consultations due to diabetes mellitus in the total observed population (separate for all age groups, children, and the elderly) ranged from 0.006 to 0.030. No statistically significant correlation for all age groups (*p* = 0.209), the elderly (*p* = 0.222), and children (*p* = 0.817) was found.

The Pearson correlation coefficient between average 24-h PM_10_ concentrations and the daily number of consultations due to diabetes mellitus in the total observed population (separate for all age groups, children, and the elderly) ranged from 0.000 to 0.036. No statistically significant correlation for all age groups (*p* = 0.126), the elderly (*p* = 0.143), and children (*p* = 0.996) was found.

The correlation analysis showed a statistically significant correlation between the daily average 24-h temperature and the daily average 24-h number concentration of UFP_0.02–0.03_ (r = −0.229, *p* ≤ 0.001), UFP_0.03–0.05_ (r = −0.295, *p* ≤ 0.001), UFP_0.05–0.07_ (r = −0.372, *p* ≤ 0.001), and UFP_0.07–0.10_ (r = −0.438, *p* ≤ 0.001), and the average 24-h concentration of PM_10_ (r = −0.482, *p* ≤ 0.001) and PM_2.5_ (r = −0.557, *p* ≤ 0.001). The correlation between the daily average 24-h temperature and the daily average 24-h number concentration of UFP_0.01–0.02_ was not statistically significant (r = −0.014, *p* = 0.577). 

The daily average 24-h number concentration of UFP_0.01–0.02_ (r = −0.131, *p* ≤ 0.001), UFP_0.03–0.05_ (r = 0.091, *p* ≤ 0.001), UFP_0.05–0.07_ (r = 0.183, *p* ≤ 0.001), and UFP_0.07–0.10_ (r = 0.249, *p* ≤ 0.001), and the average 24-h concentration of PM_10_ (r = 0.230, *p* ≤ 0.001) and PM_2.5_ (r = 0.296, *p* ≤ 0.001) have a statistically significant correlation with the daily average 24-h relative humidity. We did not confirm correlation between the daily average 24-h number concentration of UFP_0.02–0.03_ and the daily average 24-h relative humidity (r = 0.020, *p* = 0.415).

### 3.3. Relationship Analysis

The results of the non-adjusted Poisson regression models showed that the daily number of consultations due to diabetes mellitus by all age groups were statistically significantly associated with UFP_0.01–0.02_lag0_ and UFP_0.02–0.03_lag0_ (*p* ≤ 0.001) and with PM_2.5_lag2_ (*p* ≤ 0.001) and with PM_10_lag0_ (*p* ≤ 0.001). The results of the adjusted Poisson regression models showed that the daily number of consultations due to diabetes mellitus by all age groups were statistically significantly associated with UFP_0.01–0.02_lag0_ and UFP_0.02–0.03_lag0_ (*p* ≤ 0.001). More detailed information is available in Table 2.

In the observed children population, we confirmed a statistically significantly association between the daily number of consultations due to diabetes mellitus and the daily concentration of UFP_0.02–0.03_lag0_ (*p* = 0.045) and UFP_0.03–0.05_lag0_ (*p* = 0.023). The association was confirmed in case of non-adjusted Poisson regression models (Table 3). With adjusted Poisson regression models, we did not find a statistically significantly association between the daily number of consultations due to diabetes mellitus and the daily concentration of UFP, PM_2.5_ or PM_10_ (Table 3).

In the observed elderly population, we found a statistically significantly association between the daily number of consultations due to diabetes mellitus and the 24-h number concentration of UFP_0.01–0.02_lag0_ and UFP_0.02–0.03_lag0_ (*p* ≤ 0.001) and with PM_2.5_lag2_ (*p* ≤ 0.001) and with PM_10_lag0_ (*p* ≤ 0.001). The association was confirmed in case of non-adjusted Poisson regression models. The results of adjusted Poisson regression models showed that the daily number of consultations due to diabetes mellitus by the elderly population were statistically significantly associated with UFP_0.01–0.02_lag0_ and UFP_0.02–0.03_lag0_ (*p* ≤ 0.001). More detailed information is available in Table 4.

## 4. Discussion

In this study, we investigated the association between daily UFP concentrations in the ambient air and the daily number of consultations due to diabetes mellitus in primary health care units of the CHC in the MOL. The results of the study showed that the daily number of consultations due to diabetes mellitus were highly significantly associated with the daily concentrations of UFP (10 to 20 nm; *p* ≤ 0.001 and 20 to 30 nm; *p* ≤ 0.001) on the day of exposure in all age groups and in the elderly population. In the observed population of children, the association was not statistically significant. The reason for this is most probably due to the fact that children suffer mainly from Type 1 diabetes, which is genetically determined. In adults, who suffer mainly from Type 2 diabetes, lifestyle and other factors (overweight, low physical activity) play an important role in the development of diabetes. However, as already put forward, the study of the impacts of environmental factors is becoming increasingly important in the field. The associations were confirmed in adjusted models that included UFP number concentrations for five different size ranges, PM_2.5_ and PM_10_, seasonal parameters, meteorological data, and pollen data. Our findings indicated that the effects of UFP in the ambient air on the daily number of consultations due to diabetes mellitus are independent from those caused by other air pollutants, seasonal, or meteorological conditions.

Despite evidence regarding associations between PM_2.5_ and diabetes [19,20,21,22,23,24,25,26,27,28], a limited number of studies have investigated effects of UFP on diabetes mellitus [73,74,75,76]. The UFIREG study from Central Europe found associations between short-term exposure to UFP, PNC, and PM and hospital admissions caused by diabetes [73]. Due to their small size, UFP are believed to exert higher toxicity than larger particles and are associated with effects not only restricted to the lung. Three biological pathways that are assumed not to act independently are discussed. Inhalation of UFP may lead to the release of proinflammatory mediators or vasoactive molecules from lung cells causing systemic oxidative stress and inflammation, UFP deposited in the pulmonary tree may be associated with imbalance of the autonomic nervous system, and UFP and their constituents can be translocated into the blood causing endothelial dysfunction. Systemic oxidative stress and inflammation, imbalance of the autonomic nervous system, and endothelial dysfunction can lead to insulin resistance and, therefore, can promote the progression of type 2 diabetes [73]. A population-based cohort study in Toronto, Canada found each interquartile change in exposure to UFP was associated with an increased risk of diabetes [74]. One epidemiology study reported a short-term association between the prediabetic marker HbA1c and indoor, but not outdoor, UFP exposure [75]. A study of the association of long-term near-highway exposure to UFP with cardiovascular diseases, diabetes, and hypertension did not observe the association of TAA-PNC (time activity adjusted annual average UFP exposure) with diabetes [76]. These inconsistent conclusions might be explained by the subgroup of populations being targeted and the different methodologies used. The characteristic of these research studies is that they mainly covered a short observed period and the adjusted covariates were few. They examined the association between mortality and hospital admissions due to selected disease groups and UFP ambient air pollution. To our knowledge, our research is the first that examined the association between the number of first visits to the doctor at the primary health care level due to selected groups of diseases and UFP ambient air pollution. Consequently, this study addressed another important issue, namely the occurrence of less pronounced changes in population health associated with UFP ambient air pollution that do not affect mortality or require hospital admission. Further high-quality studies are needed to expand and validate the conclusions.

### 4.1. Limitations of the Study

The conducted study has some potential limitations. The possibility to establish associations between different air pollutants (including UFP) and health outcomes depends on how well the exposure can be quantified. The most obvious limitation of this study is that measured concentrations are obtained from one measuring station, which potentially creates exposure misinterpretation concerning the level of exposure for some individuals among the population. In this study, we used one single measurement site, assuming temporal variability at this site reflected the temporal variability of the exposure in the population, which was sufficient in order to estimate short-time variations in pollutant concentrations in time-series studies [77,78,79]. In addition, UFP have been shown to have a higher spatial variability than other air pollutants, therefore additional exposure misclassification might be assumed. We are aware that many factors are constantly present that affect the emissions, transformation, and dispersion of pollutants in ambient air, and thus contribute to the spatial variability that exists within urban areas. However, a study in Dresden, Germany, showed low spatial variability in UFP among urban background stations and suggested that short-time UFP exposure for the average population might be adequately represented by one measurement site if the location was chosen carefully [79]. Therefore, we assumed that the data obtained from a central measuring point, due to the location at the bottom of the MOL basin and in the city center, are a satisfactory approximation of the concentration of pollutants in ambient air for the entire urban area of MOL.

A frequent problem in using routinely collected environmental data for time-trend studies is a lack of a certain share of the measurements. In our study only a small amount of data were missing for all measured air pollutants (from 9.4% for UFP data to 3.4% for PM_10_ data). Missing data were mainly due to the calibration of instruments or filter blockage of the measuring device. 

In the toxicity of PM, the size plays a key role; nonetheless, previous studies suggested that the effects of PM on diabetes mellitus might differ with chemical composition as well [80,81,82,83]. This could be an additional limitation of the study since only the size of PM was observed, and their chemical composition was not considered. These issues certainly raise questions for future researchers on the effects of UFP on public health and suggest that forthcoming experiments should address the chemical composition of UFP. 

When using medical data, we have to take into account that the daily number of consultations due to selected diagnosis might be misestimated as well. On one hand, we assume that most subjects have a chosen personal physician near their place of residence. We are aware that this is not always the case. Therefore, individuals who have permanent residence in the MOL, but have a selected personal physician outside MOL, were excluded from the research. On the other hand, an additional limitation of such collected health data is the fact that some permanent residents of the MOL who have also a selected personal doctor there, due to daily or even weekly migrations can spend most of the time outside their place of residence, and therefore their exposure to ambient air pollutants can be different. Another limitation can be related to the relatively small population and consequently less daily number of consultations due to diabetes mellitus in primary health care units of the CHC.

As in other similar studies where data from the medical records were used, we also encountered a certain degree of data inaccuracy, which was related to coding. Some similar problems in using medical data in Slovenia were observed in a project finished in 2012 [84]. On one side, there were no problems with the completeness of data collection. One the other side, there may be differences in coding the primary diagnosis in different primary health care units of the CHC, and since only the primary diagnosis was considered, the number of cases might be misestimated.

One could argue that not all steps in a time series regression study proposed by Bhaskaran and co-authors [85] were done. However, to a certain extent we have followed these steps, while the others (a) periodic functions of time (sine/cosine functions); (b) flexible spline functions of time; (c) consider possible non-linear associations as in other regression contexts; (d) diagnostic plots based on deviance residuals; and (e) multiple sensitivity analyses changing key modelling decisions, were out of the scope of this study and were planned to be addressed in further analyses.

Despite certain limitations, this study is surely relevant, because the association between the daily UFP concentration and the daily number of consultations due to diabetes mellitus in CHC was not yet investigated in Slovenia. Further epidemiological studies are needed to establish the exact exposure assessment and to explore the impact of UFP on specific population groups, especially the more vulnerable ones and those that are more exposed.

### 4.2. Strenghts of the Study

In contrast, our study has several strengths. First, we had an extensive dataset with continuous measurements of many air pollutants and an extensive dataset about the daily number of consultations in primary health care units of the CHC Ljubljana. This allowed us to study the associations of UFP with different health outcomes in adjusted models that included UFP number concentrations for five different size ranges, PM_2.5_ and PM_10_, seasonal parameters, meteorological data, and pollen data. It was complex, extensive, and demanding research, due to the specific environment and a large number of factors that can critically affect the results of the research, and the large amount of data that needed to be processed and analyzed. Particularly it was very challenging to obtain a large amount of health data from the information system of the CHC. This action included the preparation of the command script for the extraction of data on the number of consultations, including selected diagnoses and the age of patients for a five-year period, and later the editing, cleaning, and consolidation of data by forming age classes. Large amounts of data made it possible to conclude that the effects of UFP in the ambient air on the daily number of consultations due to diabetes mellitus were independent from those caused by other air pollutants, seasonal, or meteorological conditions.

Second, the study is one of few time-trend studies in Slovenia which linked routinely collected health and environmental data with the aim of examining whether there was an association between the number of first visits to a doctor at the primary health care level and ambient air pollution [86,87,88,89].

Furthermore, this is the first study where the effects of UFP on diabetes were studied over a period of 5 years. Several studies already concluded that air pollution is likely a risk factor for diabetes. In Slovenia, as in many countries, we are seeing an increase in the prevalence of diabetes, which has become an important public health problem with accompanying morbidity and consequences for patients, families, and the community. Compared to European and OECD countries, Slovenia is in the middle zone in terms of the prevalence of diabetes. The European Health Interview Survey (EHIS) conducted in 2014 estimated the prevalence of diabetes among the adult population at 6.9%. The same result was also shown in 2016 by the Health-Related Behavioral Style Survey [90]. According to the International Diabetes Federation (IDF) Atlas, the national prevalence in adults in 2019 in Slovenia was 7.8%, and it was similar in neighboring countries (Italy 8.3%, Hungary 9.3%, Austria 9.7%, Croatia 6.8%) and Europe (8.9%) [91]. Assuming that (similarly to European countries) in Slovenia about 15% of people with diabetes are treated without medication, i.e., only with adequate physical activity, diet, and weight loss, it is estimated that in Slovenia in 2017 about 131,000 people were diagnosed with diabetes. The number of all people with diabetes (identified and undiagnosed) could range from 154,000 to 175,000 [90]. 

The results of the study could provide a valuable platform for formulating recommendations to environmental and health policymakers in Slovenia. Findings imply the adoption of an appropriate legislative framework for routine UFP measurement in the national network for ambient air quality and conveying this information to competent environmental and health institutions, and the general population. Extensive dissemination of the results and their suitable interpretation might be used for raising the awareness of the professional and lay public about the health risks associated with the presence of UFP in ambient air and the possible implementation of preventive and mitigation measures, both at the level of the population and the individual.

## 5. Conclusions

The aim of this study was to determine the association between UFP in ambient air and the daily number of consultations in the primary health care unit due to diabetes mellitus in all age groups, in children, and the elderly population of the MOL. The results of the study showed that the daily number of consultations due to diabetes mellitus were highly significantly associated with the daily concentrations of UFP (10 to 20 nm; *p* ≤ 0.001 and 20 to 30 nm; *p* ≤ 0.001) on the day of exposure in all age groups and in the elderly population. In the observed population of children, the association was not statistically significant. Our findings indicated that effects of UFP in the ambient air on the daily number of consultations due to diabetes mellitus were independent from those caused by other air pollutants, seasonal, or meteorological conditions.

The presented study addresses some important challenges in the field of public health, which have obviously been rather disregarded in view of the research carried out so far and underrated in terms of the enacted environmental health policies by Slovenia and other countries. We have established an association between the daily UFP (10 to 30 nm) concentration and the daily number of consultations for diabetes among all age groups and the elderly population. In view of that, the results of the study indicate that there is a compelling necessity for coordinated public health activities in sense of the reduction of harmful environmental factors in the MOL and the broader region. Unfortunately, the consideration of this issue in previous campaigns of the national, as well as regional or municipal, authorities has been partial and without wider socio-economic measures, such as the actions for the prevention of air pollution and the mobilization of the public for this cause.

It is clear that isolated measures of close social communities (countries, regions, cities, etc.), even if successful, will not be able to eliminate the negative effects of UFP on health. Air pollution is just one of the detrimental global consequences of uncontrolled industrialization, the lack of a sustainable model for economic development, excessive consumption, and the general ignorance of a large part of the population. All indications are that the current socio-economic arrangements concerning environmental issues urgently need radical redefinition. Some events in recent years suggest that this shift has already begun in individual segments of global society and may indicate an alternative path to less air pollution and better public health. 

## Figures and Tables

**Table 1:** Descriptive statistics for the daily number of consultations due to diabetes mellitus and ultrafine, fine, and coarse particulate matter (PM) and meteorological parameters and pollen concentration in the municipality of Ljubljana (MOL), Slovenia between 1 January 2013 and 31 December 2017.

	*n*	Mean	SD	Min.	Q_1_	Median	Q_3_	Max.
Daily number of consultations due to diabetes mellitus: all age groups, children, elderly								
All age groups	1826	11.21	9.35	0.00	1.00	11.00	18.00	42.0
Children	1826	0.04	0.21	0.00	0.00	0.00	0.00	2.00
Elderly	1826	8.17	7.19	0.00	1.00	8.00	13.00	33.00
Average 24-h number concentration of UFP (number/cm^3^)								
UFP_0.01–0.02_	1655	1230.53	478.28	16.50	915.30	1172.30	1482.50	4370.40
UFP_0.02–0.03_	1655	1086.93	434.30	88.10	809.50	1031.30	1308.20	4769.40
UFP_0.03–0.05_	1655	1517.34	605.96	247.50	1113.00	1420.30	1819.20	4446.20
UFP_0.05–0.07_	1655	1039.73	467.17	198.10	732.200	932.40	1224.20	3446.70
UFP_0.07–0.10_	1655	1070.34	578.18	181.20	696.400	924.30	1251.40	4207.90
Average 24-h concentration of PM_10_ and PM_2.5_ (µg/m^3^)								
PM_10_	1765	25.46	16.62	2.00	15.00	20.00	30.00	126.00
PM_2.5_	1742	20.67	15.25	3.00	11.00	16.00	24.00	114.00
Average 24-h of temperature (°C) and relative humidity (%)								
Temperature	1826	12.06	8.107	−7.90	5.80	12.50	18.32	30.00
Relative humidity	1826	75.99	13.66	31.00	65.00	76.00	88.00	100.00
Daily average pollen concentration (number of particles/m^3^)								
Pollen concentration	1826	8.99	27.36	0.00	0.00	1.00	7.00	323.00

Legend: *n*—total number of data; SD—standard deviation; Q1—the first quartile; Q3—the third quartile; UFP—ultrafine particles; PM_10_—particulate matter 10 micrometers or less in diameter; PM_2.5_—particulate matter 2.5 micrometers or less in diameter.

**Table 2:** Association between the daily number of consultations due to diabetes mellitus by all age groups and the average 24-h number concentration of UFP (number/cm^3^); average 24-h concentration of PM_10_ and PM_2.5_ (µg/m^3^) in the MOL, Slovenia between 1 January 2013 and 31 December 2017.

Pollutants	Model 1 ^a^				Model 2 ^b^			
	IRR	95% CI		*p*	IRR	95% CI		*p*
UFP_0.01–0.02_lag0_	1.001	1.000	1.003	<0.001	1.001	1.000	1.003	<0.001
UFP_0.02–0.03_lag0_	1.001	1.000	1.003	<0.001	1.001	1.000	1.003	<0.001
UFP_0.03–0.05_lag0_	1.000	1.000	1.000	<0.001	1.000	1.000	1.000	0.133
UFP_0.05–0.07_lag0_	1.000	1.000	1.000	<0.001	1.000	1.000	1.000	0.590
UFP_0.07–0.10_lag0_	1.000	1.000	1.000	<0.001	1.000	1.000	1.000	0.689
PM_2.5_lag2_	1.002	1.002	1.003	<0.001	1.001	1.000	1.002	0.130
PM_10_lag0_	1.002	1.001	1.003	<0.001	1.001	1.000	1.002	0.606

Legend: **^a^** non-adjusted model; **^b^** adjusted model for season of the year (spring, summer, autumn, winter), working day, influenza season, pollen concentration, air temperature, and relative humidity. IRR—incident rate ratio; CI—confidence interval; *p*—*p*-value of 0.05 or less was considered as statistically significant; UFP—ultrafine particles; PM_10_—particulate matter 10 micrometers or less in diameter; PM_2.5_—particulate matter 2.5 micrometers or less in diameter.

**Table 3:** Association between the daily number of consultations due to diabetes mellitus by the population of children and the average 24-h number concentration of UFP (number/cm^3^); average 24-h concentration of PM_10_ and PM_2.5_ (µg/m^3^) in the MOL, Slovenia between 1 January 2013 and 31 December 2017.

Pollutants	Model 1 ^a^				Model 2 ^b^			
	IRR	95% CI		*p*	IRR	95% CI		*p*
UFP_0.01–0.02_lag0_	1.000	1.000	1.001	0.164	1.000	1.000	1.001	0.663
UFP_0.02–0.03_lag0_	1.000	1.000	1.001	0.045	1.000	1.000	1.001	0.377
UFP_0.03–0.05_lag0_	1.000	1.000	1.001	0.023	1.000	1.000	1.001	0.237
UFP_0.05–0.07_lag0_	1.000	1.000	1.001	0.154	1.000	1.000	1.001	0.534
UFP_0.07–0.10_lag0_	1.000	1.000	1.000	0.676	1.000	0.999	1.001	0.873
PM_2.5_lag2_	1.004	0.988	1.018	0.571	1.004	0.985	1.021	0.668
PM_10_lag0_	1.000	0.985	1.013	0.996	0.997	0.978	1.013	0.699

Legend: **^a^** non-adjusted model; **^b^** adjusted model for season of the year (spring, summer, autumn, winter), working day, influenza season, pollen concentration, air temperature, and relative humidity. IRR—incident rate ratio; CI—confidence interval; *p*—*p*-value of 0.05 or less was considered as statistically significant; UFP—ultrafine particles; PM_10_—particulate matter 10 micrometers or less in diameter; PM_2.5_—particulate matter 2.5 micrometers or less in diameter.

**Table 4:** Association between the daily number of consultations due to diabetes mellitus by the elderly population and the average 24-h number concentration of UFP (number/cm^3^); average 24-h concentration of PM_10_ and PM_2.5_ (µg/m^3^) in the MOL, Slovenia between 1 January 2013 and 31 December 2017.

Pollutants	Model 1 ^a^				Model 2 ^b^			
	IRR	95% CI		*p*	IRR	95% CI		*p*
UFP_0.01–0.02_lag0_	1.001	1.000	1.003	<0.001	1.001	1.000	1.003	<0.001
UFP_0.02–0.03_lag0_	1.001	1.000	1.003	<0.001	1.001	1.000	1.003	<0.001
UFP_0.03–0.05_lag0_	1.000	1.000	1.000	<0.001	1.000	1.000	1.000	0.065
UFP_0.05–0.07_lag0_	1.000	1.000	1.000	<0.001	1.000	1.000	1.000	0.419
UFP_0.07–0.10_lag0_	1.000	1.000	1.000	<0.001	1.000	1.000	1.000	0.560
PM_2.5_lag2_	1.003	1.002	1.004	<0.001	1.001	1.000	1.003	0.057
PM_10_lag0_	1.002	1.001	1.003	<0.001	1.000	0.999	1.001	0.795

Legend: **^a^** non-adjusted model; **^b^** adjusted model for season of the year (spring, summer, autumn, winter), working day, influenza season, pollen concentration, air temperature, and relative humidity. IRR—incident rate ratio; CI—confidence interval; *p*—*p*-value of 0.05 or less was considered as statistically significant; UFP—ultrafine particles; PM_10_—particulate matter 10 micrometers or less in diameter; PM_2.5_—particulate matter 2.5 micrometers or less in diameter.
